# Enhanced detection of mild cognitive impairment in Alzheimer’s disease: a hybrid model integrating dual biomarkers and advanced machine learning

**DOI:** 10.1186/s12877-025-05683-5

**Published:** 2025-01-23

**Authors:** John Sahaya Rani Alex, R. Roshini, G. Maneesha, Jeetashree Aparajeeta, B. Priyadarshini, Chih-Yang Lin, Chi-Wen Lung

**Affiliations:** 1 School of Electronics Engineering, Vellore Institute of Technology, Chennai, India; 2 Department of Mechanical Engineering, National Central University, Taoyuan, Taiwan; 3 Department of Creative Product Design, Asia University, Taichung, Taiwan

**Keywords:** Mild Cognitive Impairment, Hippocampal volume, Cerebrospinal Fluid, Biomarkers, Fuzzy clustering, Hybrid learning model

## Abstract

Alzheimer's disease (AD) is a complex, progressive, and irreversible neurodegenerative disorder marked by cognitive decline and memory loss. Early diagnosis is the most effective strategy to slow the disease's progression. Mild Cognitive Impairment (MCI) is frequently viewed as a crucial stage before the onset of AD, making it the ideal period for therapeutic intervention. AD is marked by the buildup of amyloid-beta (Aβ) plaques and tau neurofibrillary tangles (NFTs), which are believed to cause neuronal loss and cognitive decline. Both Aβ plaques and NFTs accumulate for many years before the clinical symptoms become apparent in AD. As a result, in this study, CerebroSpinal Fluid (CSF) biomarker information is combined with hippocampal volumes to differentiate between MCI and AD. For this, a novel two-stage hybrid learning model that leverages 3D CNN and the notion of a Fuzzy and Machine learning model is proposed. A 3D-CNN architecture is employed to segment the hippocampus from the structural brain 3D-MR images and quantify the hippocampus volume. In stage 1, the hippocampus volume is passed through thirteen machine learning models and fuzzy clustering for classifying symptomatic AD and healthy brain (Normal Control - NC). The CSF data is fuzzified to capture the inherent uncertainty and overlap in clinical data. The identified symptomatic AD data in the stage1 are further classified into MCI and AD with the aid of a fuzzified CSF biomarker in stage 2. The experimental work presented in this study utilized the Alzheimer's Disease Neuroimaging Initiative (ADNI) dataset. The proposed hybrid model achieved an average accuracy of 93.6% for distinguishing between NC and symptomatic AD and 93.7% for discriminating between MCI and AD. This approach enhances diagnostic accuracy and provides a more comprehensive assessment, allowing for earlier and more targeted therapeutic interventions.

## Introduction

Alzheimer’s disease (AD) is a progressive neurodegenerative disease characterized by memory loss, cognitive and linguistic impairments, and behavioral alterations [1]. For diagnostic assessment, the AD progression is classified into three stages: Normal control (NC), mild cognitive impairment (MCI), and Alzheimer's disease (AD). NC represents a healthy brain. MCI is an intermediate stage identified by evident cognitive deterioration, such as increasing forgetfulness and difficulties with complicated activities, but daily life remains manageable. AD causes severe cognitive decline, including extensive memory loss, reduced daily functioning, language difficulties, and behavioral changes that frequently necessitate substantial help. The unattended AD can lead to dementia, profoundly impacting an individual's ability to function independently​ [2].

The diagnostic process for AD includes neuroimaging, standard laboratory testing, neurological examination, and a comprehensive patient history [3]. These tools collectively contribute to the assessment and understanding of the disease. Biomarkers are the measurable indications of a biological state or condition that are extracted from the assessment of imaging data and methods. Biomarkers play a pivotal role in the diagnostic process and are essential for understanding health, disease development, and therapy effectiveness. Biomarkers of Alzheimer's disease pathology can be classified into imaging and non-imaging data. Imaging modalities like Magnetic Resonance Imaging (MRI), Positron Emission Tomography (PET), and Diffusion Tensor Imaging (DTI) provide detailed insights into brain pathology. In contrast, non-imaging modality includes biospecimens (blood and cerebrospinal fluid - CSF) and clinical data (cognitive tests and genetic information). AD is marked by the buildup of amyloid-beta (Aβ) plaques and tau neurofibrillary tangles (NFTs), which are believed to cause neuronal loss and cognitive decline. In AD, both Aβ plaques and NFTs seem to accumulate for many years before the clinical symptoms become apparent [4, 5]. CSF biomarkers, particularly Aβ42, t-tau, and p-tau, are crucial for the early and accurate diagnosis of AD. They offer significant advantages in detecting AD before clinical symptoms appear, monitoring disease progression, and evaluating the efficacy of treatments [6].

In recent years, diagnosis of neurodegenerative diseases has been carried out through state-of-the-art technology such as Computer-aided diagnosis (CAD) and Artificial Intelligence (AI) tools. CAD harnesses a diverse array of brain imaging modalities to facilitate accurate diagnosis. Notably, imaging modalities show remarkable effectiveness in analyzing the structural patterns of the brain and discerning the volumetric differences between the neurotypical brain physiology of NC and AD [7]. Also, CAD utilizes significant biomarkers from neuroimages like grey and white matter volume [1, 8], hippocampus volume [9, 10] and regional cortical thickness to identify AD. The influence of imaging techniques on the detection of MCI from AD is quite limited, regardless of the number of imaging modalities investigated [11]. MCI is a challenging condition to detect because it often occurs before visible symptoms appear, making it critical to detect but elusive stage in the progression of AD. Despite these challenges, research has shown that CSF biomarkers, hippocampal volume and entorhinal volume are analyzed in identifying the progression of patients from one stage of AD to the next [12]. A study has shown that CSF biomarkers are highly effective in predicting the progression from MCI to AD. Specifically, high levels of CSF Total tau (T-tau), Phosphorylated tau (P-tau) and low levels of Amyloid β-protein (Aβ)42 can accurately identify MCI patients likely to develop AD. These biomarkers have also shown predictive value in asymptomatic elderly individuals, where reduced Aβ42 levels indicate a higher risk of developing dementia [13].

Machine learning (ML) models in AI are another alternative to CAD. By combining ML with biomarker data, researchers can identify complex patterns and relationships that would be difficult to detect using traditional methods. The standard outline followed by ML models for AD detection involves a systematic flow encompassing preprocessing, segmentation, Feature Extraction and Classification. In studies [9, 14, 15] used volume of interest (VOI) derived from MRI as a critical biomarker for AD classification. ML models like Support Vector Machines (SVM) [10, 16] and the conventional K-Nearest Neighbour (KNN) [17] have been employed as a classifier based on the VOI from MRI. However, challenges arise due to inherent uncertainties in neuroimaging data, such as noise and variations, impacting classification accuracy. To address this, researchers have incorporated fuzzy logic, which provides a framework for modeling and handling uncertainties, resulting in more robust and accurate classification [10]. ML approaches face challenges due to complex and inaccurate feature engineering, which is time-consuming and requires extensive expertise. Additionally, handcrafted feature extraction can miss important data patterns, affecting model performance and accuracy [14, 15]. On the other hand, Deep learning (DL) offers a more robust and efficient solution by automating feature extraction and learning directly from raw image data. This end-to-end approach enables DL models to capture intricate patterns and relationships within the images [18–20], surpassing the limitations of ML in terms of generalizability and accuracy. Slice wise volumetric features extracted from the Hippocampal region using a hybrid convolutional neural network (CNN) with Deep Neural Networks (DNN) effectively differentiated NC and AD [21]. For instance, DNN has achieved impressive 80–90% accuracy in differentiating between MCI and AD [22] Moreover, advanced architectures like 3D-CNN combined with stacked bidirectional long short-term memory FSBi-LSTM have demonstrated remarkable results, distinguishing AD from NC, progressive MCI (pMCI) from NC, and stable MCI (sMCI) from NC with accuracies of 94.82%, 86.36%, and 65.35%, respectively.

Early onset of AD is essential for timely therapeutic interventions, better planning and improved system management with AD [23]. When diagnosed late (after considerable cognitive decline), the efficacy of interventions is limited. Because of the subtlety of the symptoms and the lack of sophisticated diagnostic tools to detect early-stage namely mild cognitive impairment (MCI), it is often missed or misdiagnosed [24].

Current clinical practices heavily rely on cognitive tests like the Assessment Scale-Cognitive subscale (ADAS-Cog) [25] and patient history to assess the severity of cognitive symptoms in AD. However, these methods often fail to detect early disease stages. Diagnostic practices vary widely across regions due to differences in knowledge, training, and resources, hindering early and accurate AD diagnosis [23].

Our survey shows that detecting MCI is crucial for delaying the progression of AD. Combining imaging biomarkers with CSF biomarkers provides a more holistic view of the patient's condition, allowing for a better assessment of disease progression.

Our contribution is a two-stage hybrid learning model with dual biomarkers: hippocampal volume and CSF. The model's precision in identifying MCI, the critical transitional stage before AD, facilitates earlier and potentially more effective therapeutic interventions. The novelty of our work lies in utilizing 3D CNN for segmentation of hippocampus from MR imaging, fuzzy notion for removing the uncertainty in the data, and machine learning for classification which combines the strengths of each method, leading to a robust and sophisticated diagnostic tool. The sequential structure of our work unfolds with Section 2, which elaborates the methodologies of the baseline and proposed models. Sections " 3D- CNN Architecture for Segmentation" and 4 present the experimental setup and results of these models, highlighting the effectiveness of the two-stage hybrid model in detecting MCI.

## Methods and materials

### Data preprocessing

The standard data format for medical imaging research is considered in the Digital Imaging and Communications in Medicine (DICOM) format. The MR images, in DICOM format, have metadata in each slice regarding the resolution, thickness, and spacing among the slices. Any change in the metadata due to external changes or changes in the size of the patient’s brain will affect the MR image resolution. Reduction of the bias due to change in the resolution as a result of change in metadata can be achieved by 3D volume normalization and 3D volume resize. Volume normalization is a preprocessing technique commonly used in medical imaging, particularly in the analysis of 3D volumetric data from MRI or CT scans. The primary objective of volume normalization is to standardize the intensity values of voxels (3D pixels) to a consistent range, which facilitates comparison, analysis, and for further processing of the images.

The 3D volume normalization can be achieved by considering the magnitude of the voxels in the MR image given in (1).

1 $${voxel}_{r,c,h}=\frac{{voxel}_{r,c,h}-{voxel}_{min}}{{voxel}_{max}-{voxel}_{min}}$$

Where ‘r’, ‘c’, and ‘h’ denote the row, column, and height respectively. ${voxel}_{r,c,h}$ denotes the voxel magnitude at ‘r’, ‘c’, and ‘h’. ${voxel}_{min}$ and ${voxel}_{max}$ denote the minimum and maximum magnitudes of voxels in a particular 3D-MR image. This process consists of two steps: first, subtracting the minimum intensity value from each voxel's intensity, which shifts the range to start at 0, and then dividing by the range (difference between maximum and minimum values) to scale the values.

The resizing of a 3D volume using nearest-neighbor scaling involves calculating scaling factors based on the desired and current dimensions, mapping each voxel in the resized volume to the corresponding voxel in the original volume using these scaling factors, and applying the nearest-neighbor method to select the appropriate voxel value. This process ensures that the resized volume maintains the structure of the original volume while adjusting to the new desired dimensions efficiently.

The 3D MRI resizing is done with respect to its depth, breadth, and height to generate new, predetermined measurements as given in (2). To reduce the computational cost, the Nearest-neighbor image scaling method is used.

2 $${Volume}_R\;(r,c,h)=Volume\;(r\ast D_f,c\ast W_f,h\ast H_f)$$

In (2), Volume, and Volume_R_ denotes the original volume and resized volume, respectively, ‘r’, ‘c’, and ‘h’ denotes the row, column and height, respectively. Notations ${D}_{f}$, ${\text{W}}_{f}$ and ${H}_{f}$ are the scaling factors, and they are calculated as shown in (3), (4) and (5).

3 $${D}_{f}=\frac{{\text{desired}}_{\text{d}}}{{\text{current}}_{\text{d}}}$$

4 $${\text{W}}_{f}=\frac{{\text{desired}}_{\text{w}}}{{\text{current}}_{\text{w}}}$$

5 $${H}_{f}=\frac{{\text{desired}}_{\text{h}}}{{\text{current}}_{\text{h}}}$$

### Data augmentation

Data augmentation is an essential technique in ML and DL, especially when working with images. It involves creating new training samples by applying various transformations to the original data to increase the size and diversity of the dataset. This process significantly increases the size and diversity of the dataset, enabling models to generalise better and become more robust, ultimately improving their performance on unseen data. The rotate and render technique has been used to increase the training data without affecting the pixels’ resolution. This is achieved by rotating the normalized and resized MRI at six angles.

### Baseline models

This section explains the baseline models to detect the MCI with the existing architecture: 1. 3D CNN 2. Domain adaptation using transfer learning.

#### 3D-CNN Architecture for classification

CNN is a neural network architecture based on the deep learning algorithm that takes an image as an input and then converts the pixels of those images into array data types depending upon the brightness of the pixel values in the input image. The 2D CNN is suitable for 2D image features but fails to provide volumetric information. At the same time, the 3D CNN enables volumetric data analysis in medical imaging [18]. Hence, it is suitable for the analysis of 3D MR images. The advantage of 3D CNN over 2D CNN is its ability to take care of spatial and temporal information, which helps retain the changes in each consecutive frame. In 3D CNN, a 3D kernel is used to apply convolution on a stack of multiple frames, as given in (6).

6 $${\text{v}}_{\text{ij}}^{\text{xy}}= \text{ tanh} \left({\text{b}}_{\text{ij}}\text{+}\sum\nolimits_{\text{m}}{\sum }_{\text{p=0}}^{{\text{P}}_{\text{i}}\text{-1}}{\sum }_{\text{q=0}}^{{\text{Q}}_{\text{i}}\text{-1}}{\sum }_{\text{r=0}}^{{\text{R}}_{\text{i}}\text{-1}}{\text{w}}_{\text{ijm}}^{\text{pqr}}{{v}_{\left(i+j\right)m}}^{\text{(x+p)(y+q)(z+r)}}\right)$$

Where, ${{\text{P}}_{{\varvec{i}}}{,\text{Q}}_{{\varvec{i}},}\text{R}}_{{\varvec{i}}}$ denotes the dimensions of the 3D kernel of the i^th^ layer, ${\text{w}}_{\text{ijm}}^{\text{pqr}}$ 10

**Fig. 1:**
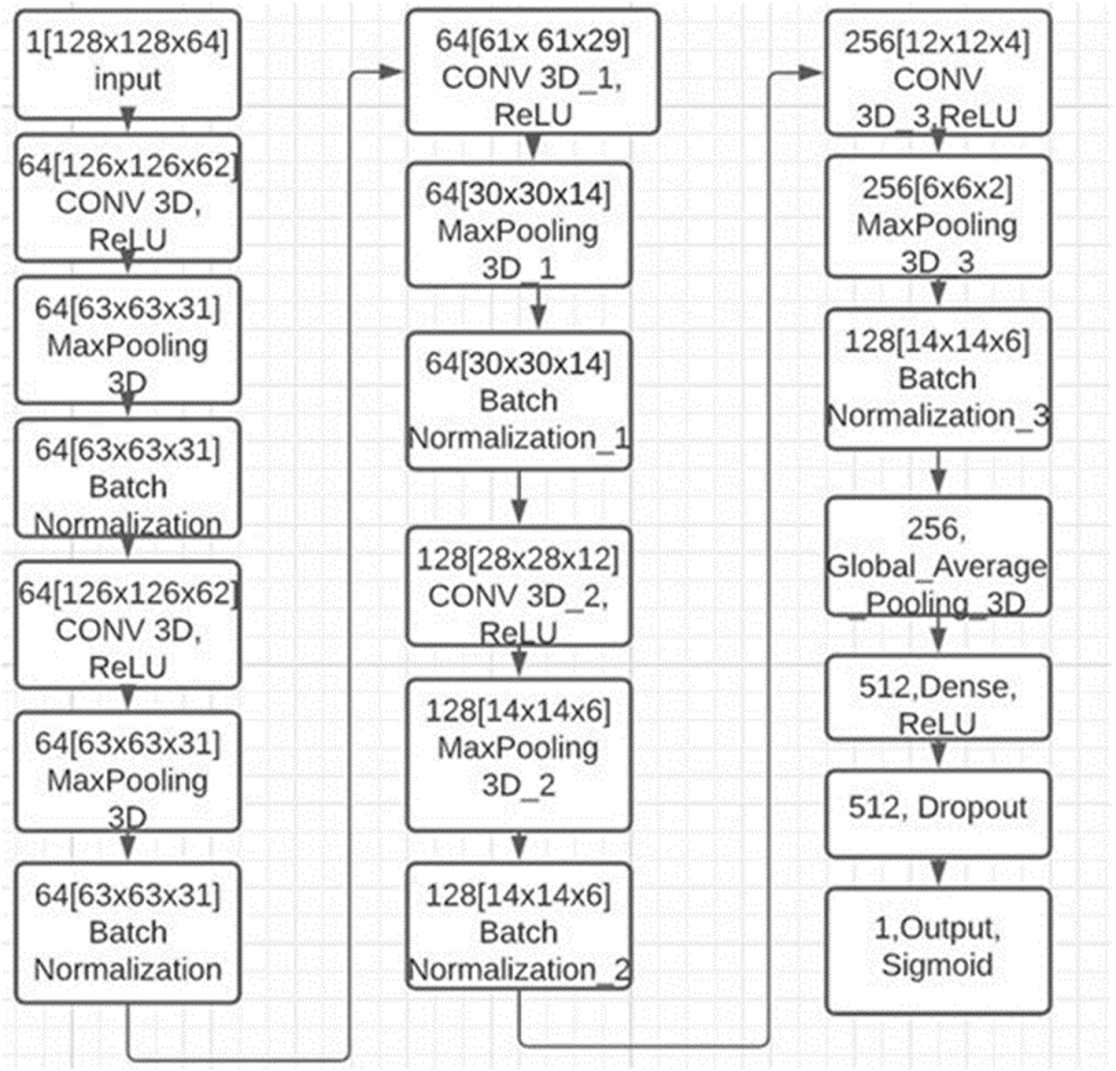
3D CNN Architecture for Classification

${{v}_{\left(i+j\right)m}}^{\text{(x+p)(y+q)(z+r)}}$ the input feature map value at the spatial location (x+p, y+q, z+r).${\text{V}}_{\text{ij}}^{\text{xy}}$ denotes the output feature map of a i^th^ layer and ${b}_{ij}$ denotes the bias [18]. A 17-layer, 3D CNN is designed for 3D MR image classification. This architecture has 1,352,897 trainable parameters and it is shown in Fig. 1.

#### 3D-CNN Architecture for Segmentation

**Fig. 2:**
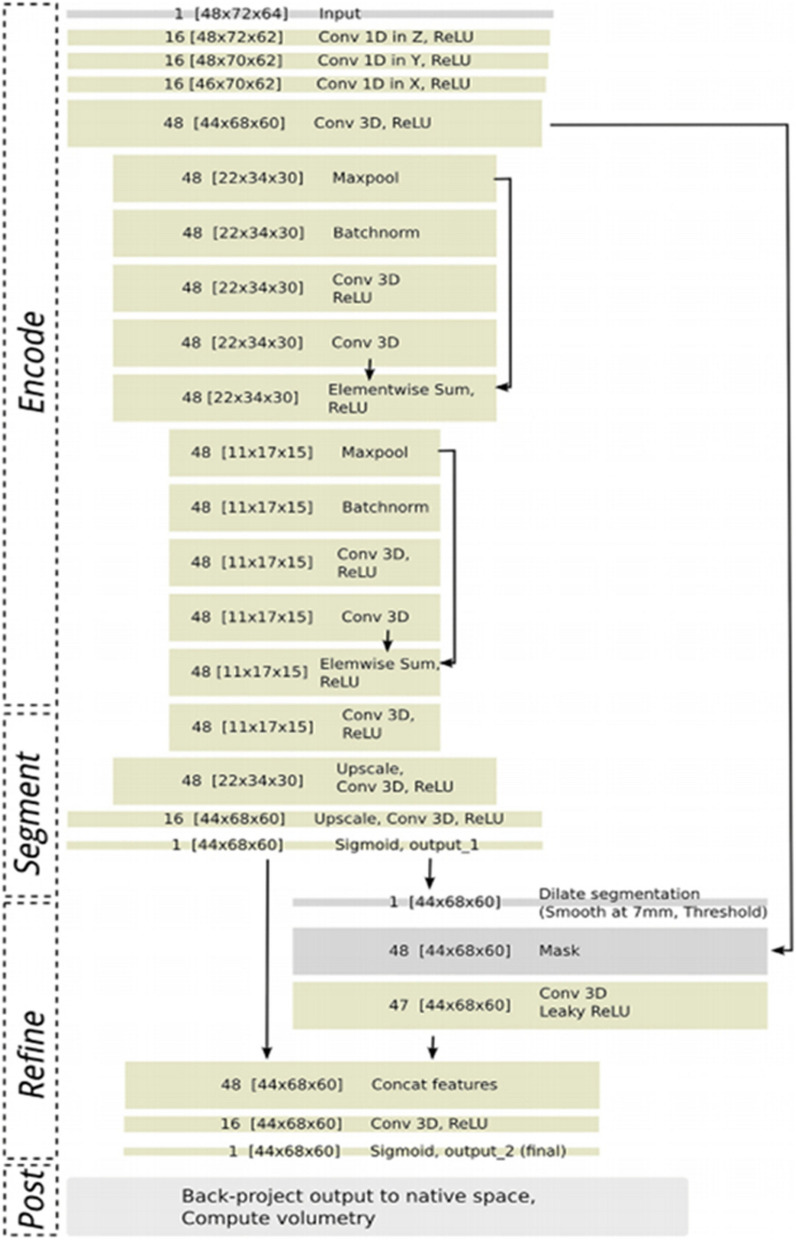
3D CNN Architecture for segmentation of the hippocampal region [26]

The 3D Deep CNN [26] adopted for segmenting the region of interest, which is the hippocampal region of the brain. As the volume of the hippocampus decreases, it indicates the progression of disease where the images are fed, and we obtain the output that is 2 segmented .nii file that contains the left and right hippocampal regions. This architecture consists of encoder block, segment block, refine block and post processing where the volume is calculated. The ConvNet outputs a segmentation map that finds the probability of a voxel in the segmented part that is projected back to the native space and is optionally thresholded. The volume of the hippocampus is then calculated from this segmented .nii file. Figure 2 depicts the entire process of segmentation and volume calculation. This segmented hippocampus voxel is given to the discussed 3D-CNN classifier in 2.3.1 to classify NC, AD, and MCI.

### Domain adaptation

In general, image classification using deep learning requires a large set of annotated data to train the model for classification in the supervised learning paradigm. However, not all the applications have massive annotated datasets. It requires intense manual labour, so a domain adaptation is followed for biomedical image classification. Transfer learning is a technique that involves retaining knowledge, such as features and weights, from a pre-trained model and using it to train the pretrained models on different datasets. It saves time and computing resources by applying the pre-trained model’s learned features and weights. Transfer learning domain adaptation is particularly beneficial in medical imaging, especially when dealing with small datasets [27]. This work considers three *Keras* pre-trained deep learning models, namely, MobileNetV1, InceptionNetV2, and DenseNet201. These models were pre-trained with the ImageNet dataset. The domain adaptation process begins with raw 3D MRI data converted into 2D images to classify subjects as NC, AD, or MCI.

### Proposed method

**Fig. 3:**
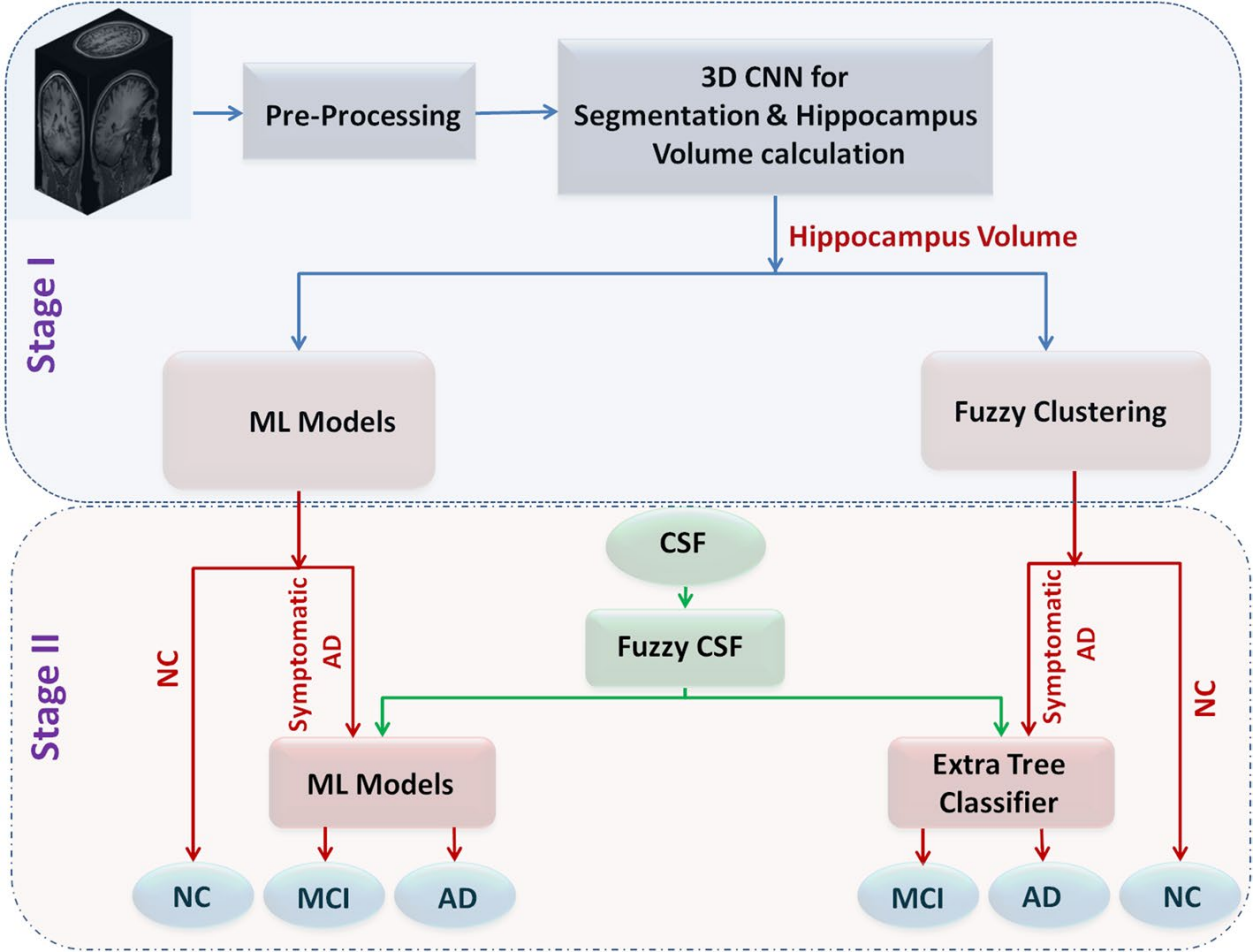
Proposed Models

AD is characterized by various clinical symptoms, with hippocampal atrophy being a prominent feature. The decrease in hippocampal volume serves as a reliable indicator of the progression of the disease. Two novel hybrid learning models are proposed, each encompassing two stages: (1) Classification of NC versus Symptomatic AD, and (2) Classification of MCI versus AD using the Symptomatic AD data identified in stage 1. These models are illustrated in Fig. 3.

#### Proposed Hybrid Learning Model 1 (HLM1)

In the hybrid learning model 1 (HLM1), 3D CNN architecture explained in section 2.3.2 segments the hippocampal voxel is segmented, and volume is quantified. Using the obtained hippocampal volume, the machine learning models classify the subject as either NC or symptomatic AD. In stage 2, the symptomatic AD data were further distinguished into MCI and AD using CSF biomarkers of symptomatic AD. To address the uncertainty present in the CSF biomarkers, they undergo a fuzzification process. The notion of fuzzy is used for handling uncertainty and imprecision, which is particularly useful in medical diagnoses where biomarker values can overlap between different conditions like AD and MCI. The primary difference between the fuzzy set and the traditional set is the way they use the set elements to make any decision. In traditional logic, an element either belongs to a set or does not (e.g., a patient either has AD or does not) depending on a threshold, whereas fuzzy logic introduces the concept of partial membership where each element (or patient) belongs to both the sets (AD or not) with a certain degree of membership. Each biomarker value is assigned a membership function, which quantifies the degree to which that value belongs to a particular set. To incorporate the concept of fuzzy, each biomarker value is assigned a membership function, which quantifies the degree to which that value belongs to a particular set (e.g., AD or MCI). Whenever biomarkers for AD and MCI overlap, fuzzy logic helps in managing this overlap by assigning degrees of membership rather than forcing a traditional logic. This is crucial because, as AD progresses, some biomarkers increase while others decrease, and a strict threshold could misclassify the patients. The fuzzy membership for each biomarker is calculated as given in (7).

7 $${\mu }_{ij}=\frac{1}{{\sum }_{k=1}^{C}{\left(\frac{\Vert {x}_{j}-{c}_{i}\Vert }{\Vert {x}_{j}-{c}_{k}\Vert }\right)}^{\frac{2}{m-1}}}$$

Where, ${\mu }_{ij}$ denotes the fuzzy membership of the j^th^ biomarker towards the i^th^ cluster center, x_j_ denotes the j^th^ biomarker, c_i_ and c_k_ denotes the i^th^ and k^th^ cluster center respectively, and m denotes the fuzziness parameter.

This membership function is derived from the Fuzzy c-mean objective function given in (8).

8 $$J\left({U}_{ij}, {C}_{i}\right)=\begin{array}{c}Minimize\\ {U}_{ij}, {C}_{i}\end{array}\sum\nolimits_{i=1}^{2}\sum\nolimits_{j=1}^{n}{{\mu }_{ij}}^{m}{\Vert {x}_{j}-{c}_{i}\Vert }^{2}$$

**Fig. 4:**
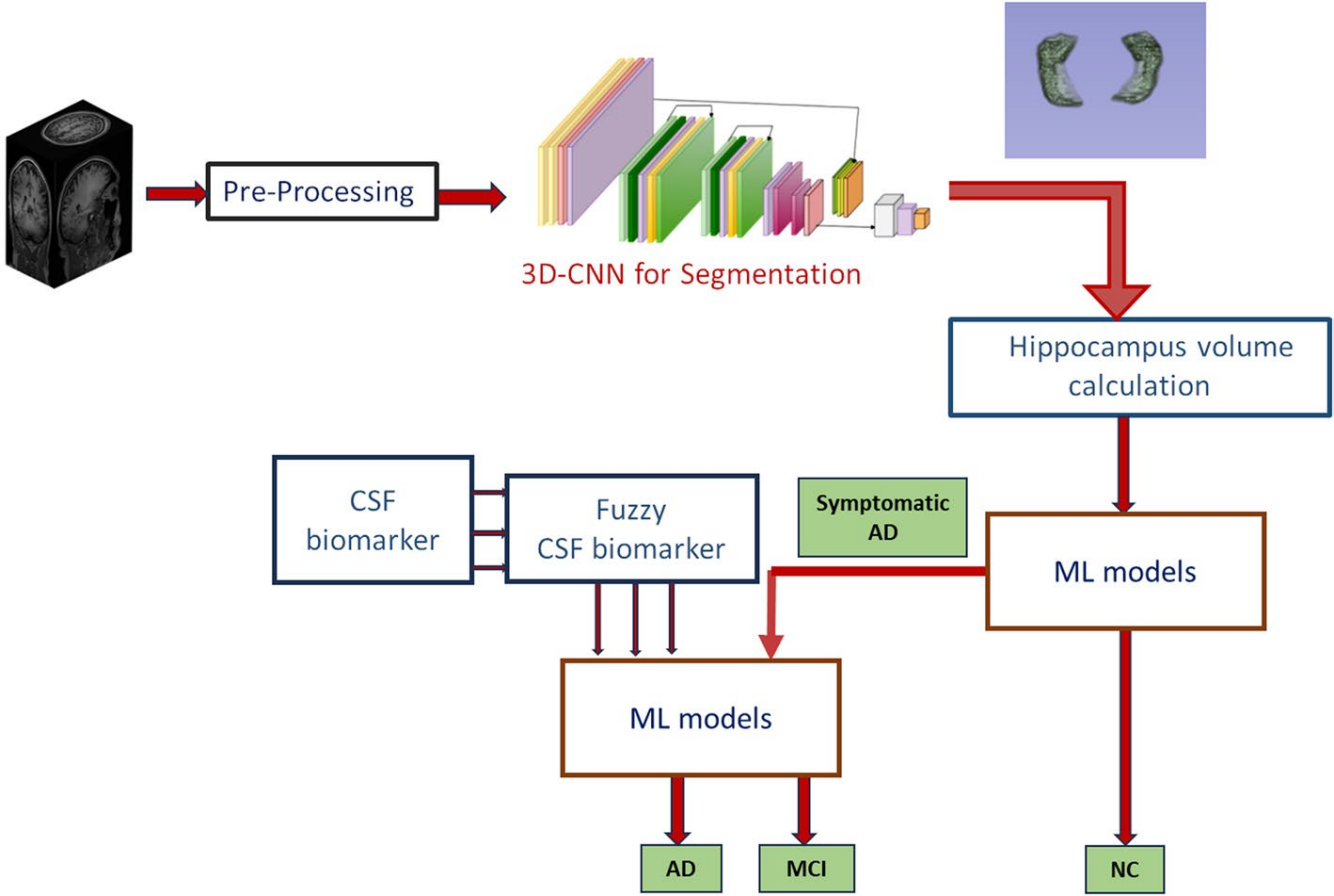
Proposed HLM1

Subject to condition: $\sum_{i=1}^{2}{\mu }_{ij}=1, j=1, 2, \dots , n$

where, J is minimized to estimate the best cluster centres c_i_. c_i_ denotes the cluster centres of the cells belonging to AD and MCI, ${\mu }_{ij}$ denotes the fuzzy membership of the j^th^ biomarker towards the ith cluster centre, and n denotes the total number of biomarkers. Thus, hippocampal volume and fuzzified CSF biomarkers are given as input to the machine learning models for MCI detection. The same is explained through the pseudocode as shown in Algorithm 1. Figure 4 shows the block diagram of HLM1

**Figure:**
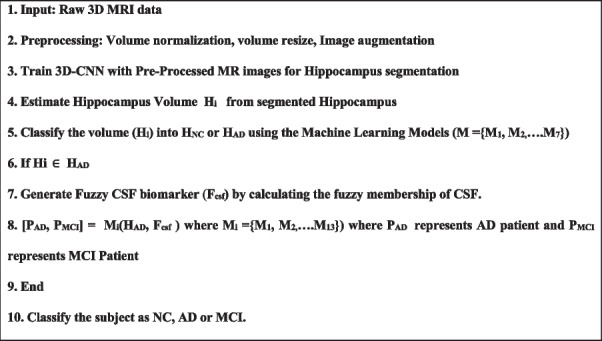
** Algorithm 1 - Hybrid Learning Model 1**

In the above algorithm, M_1_, M_2._.. M_13_ are the ML models, namely SVC, Decision tree, Random forest, XGB, LGBM, Extra Tree, Gradient Boosting, Ada Boost, K Neighbors, MLP, Gaussian NB, Logistic and Voting classified (soft).

#### Proposed Hybrid Learning Model 2 (HLM2)

**Fig. 5:**
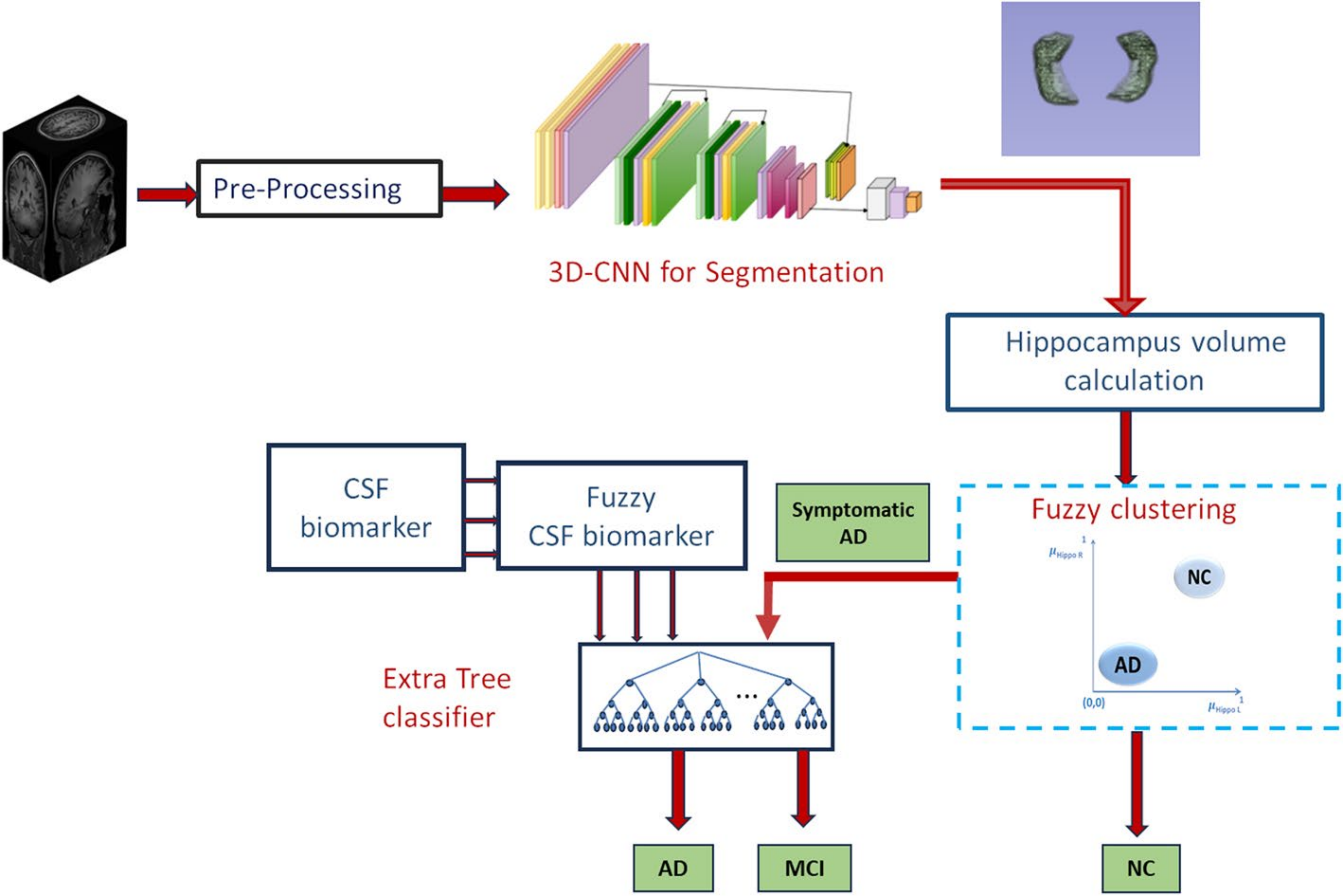
Proposed HLM2

In the hybrid learning model 2 (HLM 2), the hippocampus segmentation is obtained using a 3D CNN architecture explained in section 2.3.2, and the volume is quantified. The volume obtained might have uncertainty during the segmentation and quantification. To address this unpredictability, fuzzy clustering is used to determine whether the volume corresponds to AD or NC, rather than relying on a machine learning model. Figure 5 shows the block diagram of HLM2. In HLM 1, it has been observed that high accuracy is obtained by the Extra Tree classifier while classifying NC vs. AD. So, in stage 2, the identified symptomatic AD subjects are further classified into MCI or AD with the help of the fuzzified CSF by Extra Tree classifier model. The same is explained through the pseudocode as shown in Algorithm 2. Figure 5 shows the block diagram of HLM2.

## Experimental setup

**Figure:**
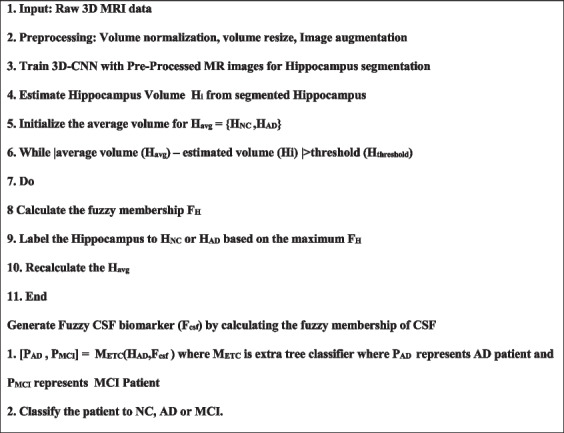
** Algorithm 2: Hybrid Learning Model 2 (HLM2)**

### Experimental setup

#### Dataset

All experiments were conducted using the Alzheimer’s Disease Neuroimaging Initiative (ADNI) clinical dataset (https://adni.loni.usc.edu). The ADNI repository includes imaging, clinical, and genetic data for over 2,220 patients across four studies (ADNI1, ADNI2, ADNI GO, and ADNI3). These datasets differ in demographics and medications and include periodic follow-ups of patients. Imaging data from ADNI1, ADNI2, and ADNI GO comprises MRI and PET scans. The data publisher has standardized these images to mitigate nonlinearity caused by scanners from different vendors. For this study, 630 cross-sectional 3D T1-weighted MRI images were used, each containing 9,108 voxels distributed across 18 slices (1.2 mm thick), with 22–23 voxels per slice. Additionally, 500 CSF data samples from ADNI1, ADNI2, and ADNI3 were included in the analysis. AD and MCI MR images were selected based on the availability of the CSF data. The Demographic details of the subjects are given in Table 1.

**Table 1:** Demographic details of the subjects

**Group**	**No of Subjects**	**Male**	**Female**	**Age Range**
AD	215	133	82	55–93
NC	204	75	129	57–95
MCI	211	123	89	58–94

#### Baseline model configuration

##### 3D-CNN Architecture for classification

Dataset of 630 3D MRI images was applied via the data augmentation method resulting in 3780 3D MRIs being considered for 3D CNN classification. A custom built 17 layered 3D-CNN model with 1,352,897 parameters as explained in section 2 with 3 class softmax layer is designed for classification. *Adam* optimizer is applied, and Softmax activation function is used for the output layer and *ReLU* for other layers. The ratio of train and validation data is 9:1. The segmentation of the hippocampal region from 3D MRI data is done using a hippo deep network. This hippo deep network is trained using segmented hippocampal images obtained from Free Surfer. The size of those images is [432, 288]. A total of 630 3D-hippocampus were segmented from 3D MRI and applied to a custom-built 17-layer 3D CNN model for classification. The dataset was split into a training set and a validation set with a ratio of 9:1.

##### Domain transfer

According to the literature, the hippocampus region is visible in only a limited number of slices in the coronal view. Here, 630 3D MRI scans were taken from ADNI and 18 slices of 2D coronal view images were taken from each 3D MRI scan resulting in a total of 11340 images. Typical *Keras* pretrained model takes an input of image size 224 x 224. So, resizing of the 2D images has been performed using med2image, a simple utility in Python that converts medical files to .jpg or .png format to match the requirement of the pre-trained deep learning models. The last layer of the pretrained model has 1000 classes. But, for our application, the SoftMax layer is configured with three classes (NC, MCI, AD) classification. The network is optimized with RMSProp and ADAM optimizers. Both the optimizers reported similar performance, with RMSProp giving slightly better accuracy. The learning rate was set to 0.001. The model was trained for 150 epochs for each of these models and fine tuned from 150-th epoch for 50 more epochs. Each of these pretrained models are finetuned from 3/4th of the total layers in each model.

#### Proposed model setup

A dataset of 630 original 3D MRIs is considered for the proposed model. During the second stage, symptomatic AD cases with 500 CSF data points were included. The dataset was split into a training set and a validation set with a ratio of 7:3. During training, an initial learning rate of 0.0001 was set, and a learning rate schedule based on exponential decay was employed to adapt the learning rates. The model was compiled using binary cross-entropy loss and optimized with the Adam optimizer. The training process was conducted for 100 epochs. The experiments were conducted on the GoogleColab platform, utilizing its GPU resources for efficient model training and evaluation.

## Results and discussion

### Baseline model results

#### 3D CNN Architecture for classification

**Fig. 6:**
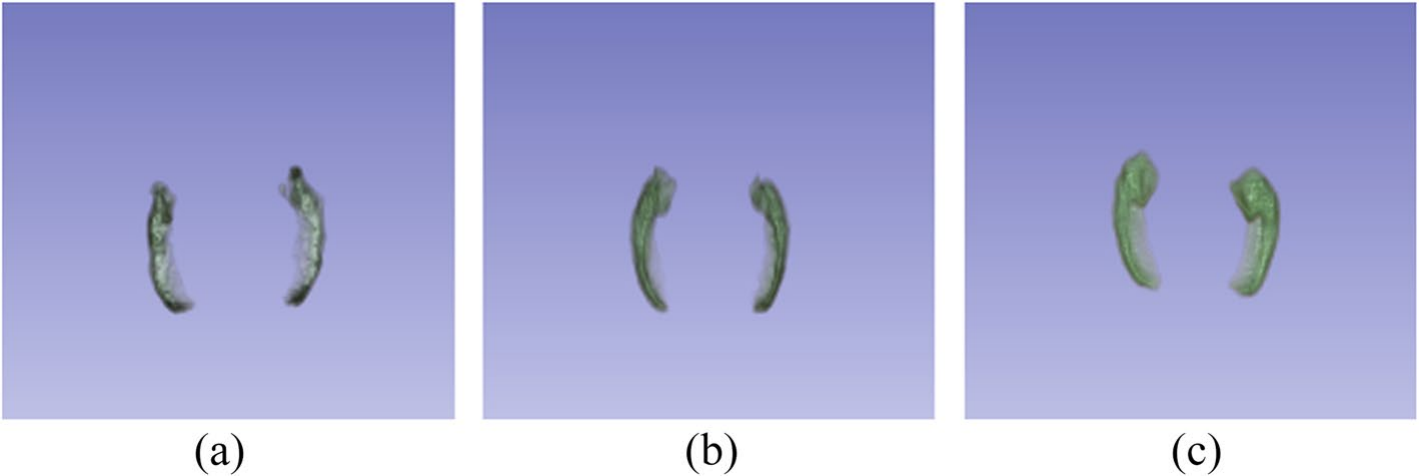
Segmented left and right hippocampal region (**a**) AD, (**b**) MCI, and (**c**) NC

The segmented left and right hippocampal atrophy for each AD, MCI, and NC are shown in Fig. 6. As we can see the thickness of the hippocampal region becomes thin as it transitions from NC to AD via MCI

As discussed, the 17-layered 3D CNN model in section 2.3.1 is employed to classify the original 3D MR images and the voxels from the segmented hippocampus image. The experimental results show that using the segmented hippocampus from MRI for classification significantly improves accuracy, achieving a 10% increase compared to using the original 3D MRI data. This result indicates that incorporating hippocampal segmentation improves the classification model's performance, enhancing its ability to accurately assign MRI data to their respective classes. Table 2 shows the Classification accuracy of the 3D CNN architecture.

**Table 2:** Accuracy of the 3D CNN architecture

**Method**		**Accuracy**
3D CNN Classification	Original MRI	44.1%
	Segmented hippocampus	54%

#### Domain transfer

The *Keras* models' hyperparameters are around 3 million for MobileNet, 50 million for InceptionNet, and 20 million for DenseNet. In terms of the number of layers, MobileNet has 86, while InceptionNet and DenseNet have around 700. The highest validation accuracy obtained is 67.7% for MobileNet with fewer trainable parameters. The other architectures, namely InceptionNet and DenseNet, yielded 58.6% and 58%, respectively; the same is shown in Table 3. MobileNetV1 is a lightweight architecture that has depthwise convolution and pointwise convolution, which makes it better than other models. Still, the domain adaptation method did not yield good performance even though the networks were fine tuned for MR images.

**Table 3:** Comparison of accuracy of domain transfer models

**Model**	**Parameters**	**No of layers**	**Accuracy**
MobileNetV1	3,231,939	86	67.7%
InceptionNetV2	54,336,736	708	58.6%
DenseNet201	18,327,747	707	58%

### Proposed model result

#### Hybrid learning model 1

**Fig. 7:**
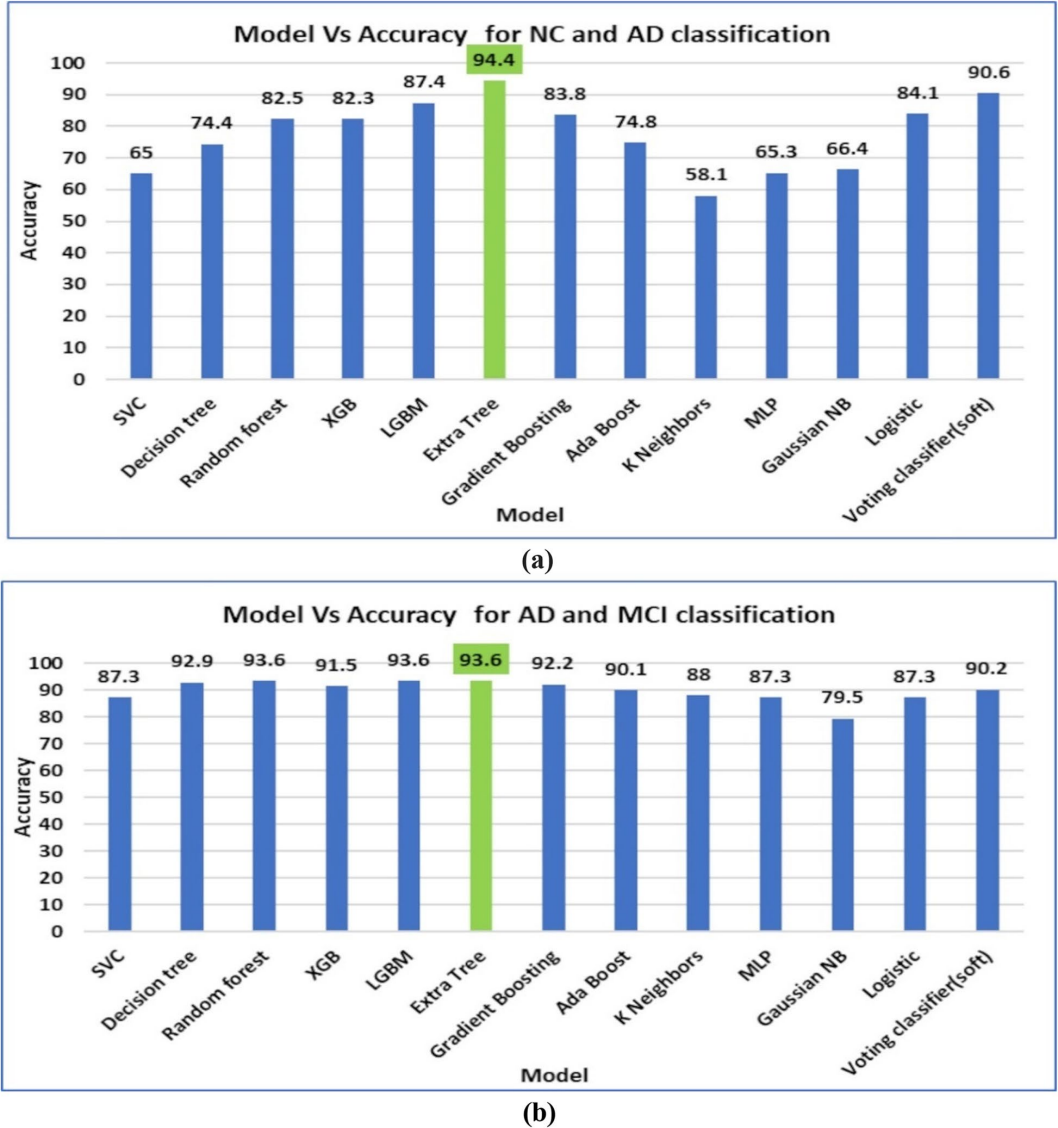
Model Vs Accuracy of HLM1 (**a**) stage 1 (**b**) stage 2

In HLM1, the left, right, and total hippocampal volumes are taken as input to the ML models for classifying the subject as NC or symptomatic AD. Thirteen machine learning models, namely Support Vector Classifier, Random Forest, eXtreme Gradient Boosting, Light Gradient Boosting, Extra Trees, Gradient Boosting, Ada Boost, K-Nearest Neighbors, Multi-Layer Perceptron, Gaussian Naive Bayes, Logistic Regression, and a Voting Classifier are trained to classify the quantified hippocampal volume into NC or AD. Among them, the Extra Trees Classifier yielded a high accuracy of 94.4%. The Extra Trees Classifier is an ensemble method that constructs multiple unpruned decision trees with random attributes and cut-point selection, increasing diversity and reducing overfitting. This randomness enhances computational efficiency and creates a robust, generalisable model by aggregating de-correlated trees through majority voting. Consequently, it captures various data patterns more effectively than individual decision trees [28]. Further, in stage 2, fuzzified CSF biomarkers like Abeta, T-tau, P-tau and CSF ratio are taken along with the hippocampal volume biomarkers; the ML models achieved a high accuracy of 93.6% for classifying AD and MCI. These results prove the effectiveness of the HLM1 approach using dual biomarkers. The efficacy of the usage of dual markers is also discussed in the ablation study. To provide a comprehensive overview of the results, the accuracy obtained using various ML models has been listed in Table 4, and the same is corroborated by the bar plot shown in Fig. 7a and b.

**Table 4:** Accuracy of the proposed HLM1

	***STAGE 1*** 3D CNN segmentation+ ML models (NC vs. symptomatic AD)	***STAGE 2*** ML models fuzzified CSF (MCI vs. AD)
SVC	65	87.3
Decision tree	74.4	92.9
Random forest	82.5	93.6
XGB	82.3	91.5
LGBM	87.4	93.6
Extra Tree	94.4	93.6
Gradient Boosting	83.8	92.2
Ada Boost	74.8	90.1
K Neighbors	58.1	88
MLP	65.3	87.3
Gaussian NB	66.4	79.5
Logistic	84.1	87.3
Voting classifier(soft)	90.6	90.2

#### Hybrid learning model 2

In HLM2, instead of ML models, to manage the uncertainty of the quantified hippocampal volume, fuzzy clustering was implemented to classify NC or symptomatic AD in stage 1. Based on the best performance of the Extra Tree Classifier from HLM1, it is selected as the classification model for HLM2 in stage 2. The experimental results indicate an impressive accuracy of 92.8% for distinguishing between NC and symptomatic AD and 93.8% for MCI and AD. These results highlight the potential of HLM2 to distinguish between MCI and AD, thereby facilitating a more accurate and effective diagnosis. Table 5 shows the accuracy obtained using HLM2. Considering both HLM1 and HLM2, the average accuracy achieved is 93.6% for NC vs. symptomatic AD and 93.7% for distinguishing between MCI vs. AD. These average accuracy values demonstrate the consistent performance of the hybrid learning models in accurately classifying MCI vs AD of the MRI data.

**Table 5:** Accuracy of the HLM2

	***Stage 1*** 3D CNN segmentation + fuzzy clustering (NC vs symptomatic AD)	***Stage 2*** Extra tree classifier +fuzzified CSF (MCI vs. AD)
Accuracy	92.8	93.8

### Comparison of our experimental results

A comparison of baseline and proposed model accuracy is shown in Table 6. Traditional 3D CNN model with 17 layers has trainable 1,352,897 parameters. This requires a huge amount of data for training and also high-end computing systems with large amounts of GPUs. With 4000 3D-MRI, 3D-CNN model accuracy yielded 50 to 60%. Next, we have utilized pre trained *Keras* models using domain adaptation. Though the data set is 11340, still the classification accuracy is poor in domain adaptation. This is because the pretrained models are trained on ImageNet dataset, which does not share any high-level feature with brain images. Instead of 3D-CNN for classification, in our proposed hybrid models, we have leveraged 3D-CNN to segment the Region of Interest (ROI)-hippocampus and quantify the volume. This hippocampus volume biomarker is fed to machine learning classifiers which performed much better than deep learning models with smaller data. Also, in stage1, the Fuzzy clustering was employed to handle the inherent uncertainty and overlap between different stages of cognitive impairment. By assigning membership probabilities rather than hard labels, our model in stage-1 can more accurately distinguish between normal controls (NC) and symptomatic AD, especially in scenarios with limited data points. Integrating the hippocampal volume with the fuzzified cerebrospinal fluid (CSF) biomarkers allows our model in stage-2 to leverage complementary information from both imaging and biochemical data. Our proposed models HLM1 and HLM2 yielded accuracy of 93.8% and 93.6 % in distinguishing MCI and AD.This holistic approach enhances diagnostic accuracy by providing a more complete picture of the patient’s condition, making it easier to distinguish between MCI and AD, even with a limited number of samples.

**Table 6:** Comparison of proposed models vs baseline models

**Method**	**Classification**	**Accuracy**
3D CNN Classification	NC vs MCI vs AD	54%
MobileNetV1	NC vs MCI vs AD	67.7%
HLM1	NC vs Symptomatic AD	94.4%
	MCI vs AD	93.6%
HLM2	NC vs Symptomatic AD	92.8%
	MCI vs AD	93.8%

The proposed model can be used as an effective preliminary screening tool, especially in high-patient volume settings. It identifies individuals at risk for Mild Cognitive Impairment (MCI) or Alzheimer's Disease (AD) by analyzing their Hippocampal volume and cerebrospinal fluid (CSF) data. By focusing on these at-risk individuals, healthcare providers can prioritize them for further clinical evaluation, enabling timely interventions. This approach improves outcomes for people experiencing cognitive decline by reducing the evaluation process and increasing the likelihood of early treatment.

### Comparison with existing literature work

The effectiveness of our proposed methodology is assessed with the results of existing literature, which is shown in Table 7. All the literatures shown in Table 5 used ADNI dataset. In [29], CNN was trained with MRI image patches for classifying NC and AD. The size of the dataset utilized is 818 MRI images. Kanghan et al. [18] used convolutional autoencoder (CAE) based unsupervised learning for the AD vs. NC classification task with 694 MRI scans. On the other hand, the proposed hybrid model achieves better accuracy with a smaller dataset of 630 MRI images with 500 CSF. It outperforms a DNN [22] used to classify NC vs. MCI vs.AD

**Table 7:** Performance comparison of proposed hybrid models with existing literature on ADNI dataset

**Authors**	**Methods**	**Dataset**	**No of Images**	**Accuracy in %**
Weiming Lin et al. [29]	CNN	AD vs NC	818	88.79
Kanghan et al [18]	Convolutional Autoencoder +Transfer learning	AD vs NC	694	86.60
P C Muhammed Raees et al [22]	DNN	NC vs.MCI vs.AD	-	80–90
**Proposed HLM1**	3D CNN→ML Model [stage 1] ML Model [stage 2]	NC vs Symptomatic AD MCI vs AD	630	**94.4** **93.6**
**Proposed HLM2**	3D CNN→Fuzzy Clustering [stage 1] Extra Tree Classifier [stage 2]	NC vs Symptomatic AD MCI vs AD	630	**92.8** **93.8**

### Ablation study

**Fig. 8:**
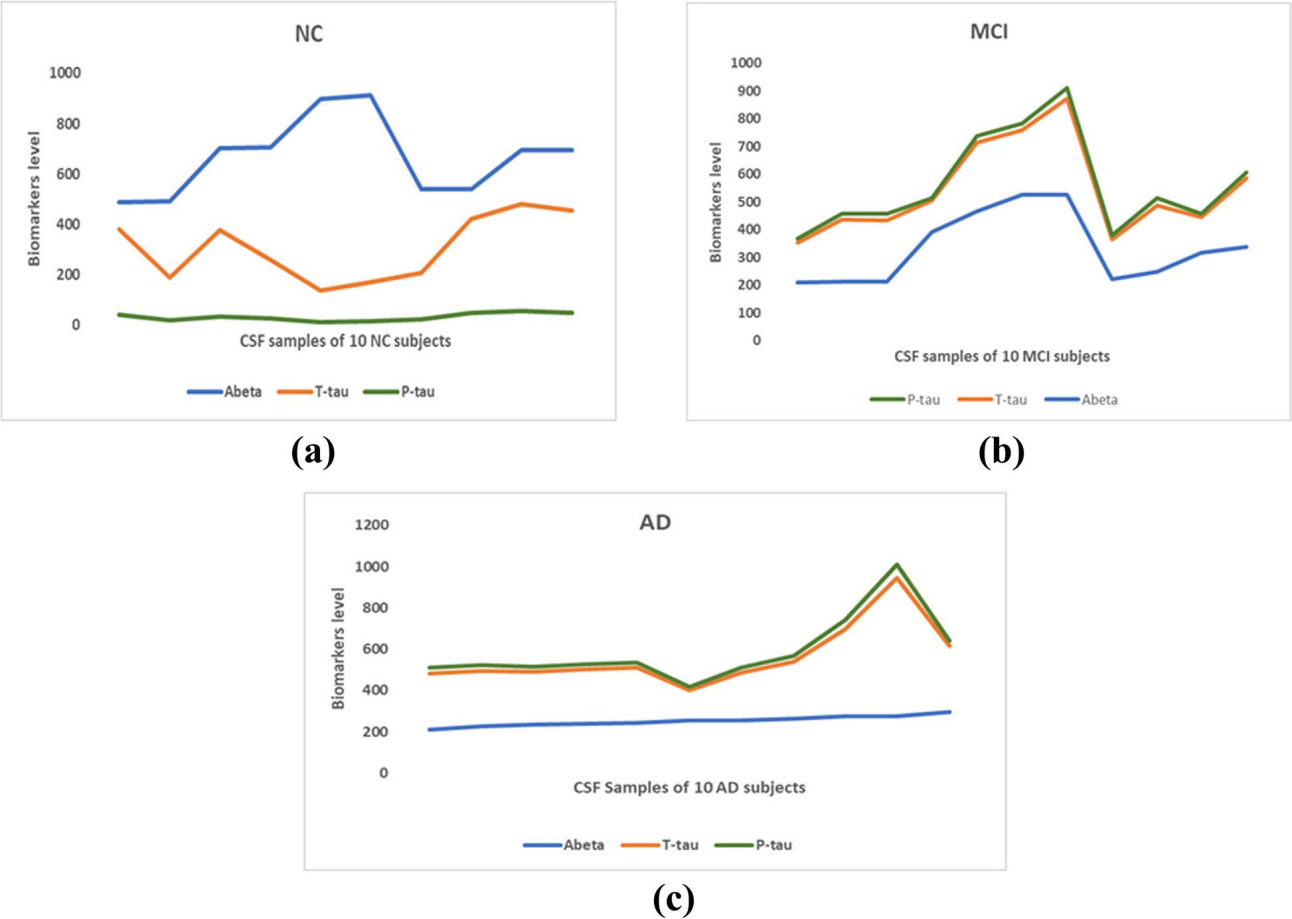
CSF biomarkers: Abeta, T-tau, P-tau distribution in (**a**) NC, (**b**) MCI, and (**c**) AD

**Fig. 9:**
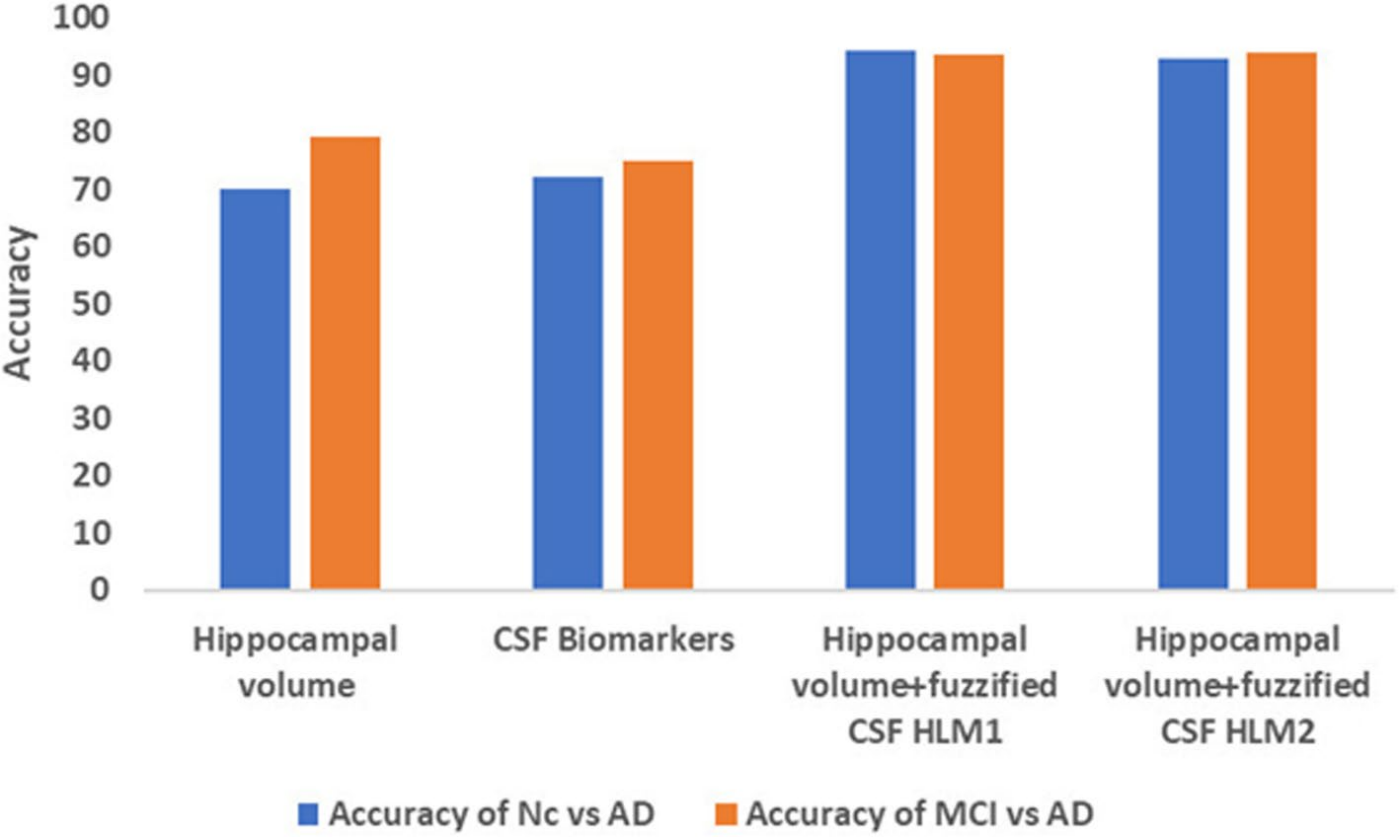
Influence of each biomarker in detecting MCI

This study leverages dual biomarkers-hippocampal volume and CSF, for classifying individual subjects into NC, MCI, and AD. Figure 8a, b, and c show the CSF distributions of 10 subjects in each of the three groups: NC, MCI and AD. These distributions reveal distinct patterns in the levels of the Abeta, T-tau, and P-tau biomarkers. In individuals with AD, there is a noticeable trend of decreasing Abeta levels, accompanied by increasing T-tau and P-tau levels. Conversely, the NC group typically exhibits normal or higher Abeta levels and lower T-tau and P-tau levels. A decrease in Abeta levels is observed for individuals with MCI; Additionally, T-tau and P-tau levels in MCI are higher than in the NC group but lower than in the AD group. This finding suggests a potential transitional state, as mentioned in reference [20]. Using the hippocampal biomarker alone as input to the extra tree classifier yielded 70% accuracy for NC vs. AD and 79% for MCI vs. AD, respectively. In another experiment, CSF biomarkers alone were used as the input to the extra tree classifier, which resulted in an accuracy of 72% and 75% for NC vs. AD and MCI vs. AD, respectively. These results indicate that the approach of using dual biomarkers and the fuzzification process proved to be more effective in accurately classifying individuals into the respective groups. The impact of each biomarker in detecting MCI is given in Fig. 9 by a bar plot. The limitations of this study are primarily due to the small dataset size, which is a result of the scarcity of subjects with both MRI and cerebrospinal fluid (CSF) data. The limitations of this study are primarily due to the small dataset size, which arises from the scarcity of subjects with both MRI and cerebrospinal fluid (CSF) data. This limitation significantly restricts the model's effectiveness. Further, the Deep learning model requires a high-end computing system with more than 48GB -GPU memory to train the model for better accuracy with more data. Currently, we have trained our models with GoogleColab. Accuracy will be improved when the models are trained with more data.

## Conclusion

Alzheimer's disease is a complex neurological condition characterized by progressive cognitive decline, which can lead to dementia if not diagnosed and managed in its early stages. Early diagnosis and intervention are crucial for slowing the progression of the disease and improving the quality of life for affected individuals. In this research, hybrid models are presented to detect MCI. The integration of multiple data sources (MRI and CSF) from ADNI provides a more holistic view of the patient's condition, allowing for a better assessment of disease progression. The model's precision in identifying MCI, the critical transitional stage before AD, facilitates earlier and potentially more effective therapeutic interventions. The proposed hybrid approach allows for more nuanced differentiation between different stages of cognitive impairment, which is crucial for tailoring treatment plans to individual patients. The model can be adapted and scaled to incorporate additional biomarkers and imaging techniques, making it flexible for future advancements in AD research and diagnosis.

## Data Availability

The data used in this study is available in Alzheimer's disease Neuroimaging Initiative: ADNI.
